# Potential of atmospheric pressure ionization sources for the analysis of free fatty acids in clinical and biological samples by gas chromatography-mass spectrometry

**DOI:** 10.1007/s00216-022-04223-z

**Published:** 2022-07-18

**Authors:** Paul E. Görs, Pia Wittenhofer, Juan F. Ayala-Cabrera, Sven W. Meckelmann

**Affiliations:** Applied Analytical Chemistry, University of Duisburg-Essen, Universitätsstrasse 5, 45141 Essen, Germany

**Keywords:** Non-esterified fatty acids, Fatty acids, Gas chromatography, Pentafluorobenzyl bromide, Systems biology, Lipidomics

## Abstract

Because of the central role of fatty acids in biological systems, their accurate quantification is still important. However, the impact of the complex matrix of biologically and clinically relevant samples such as plasma, serum, or cells makes the analysis still challenging, especially, when free non-esterified fatty acids have to be quantified. Here we developed and characterized a novel GC–MS method using pentafluorobenzyl bromide as a derivatization agent and compared different ionization techniques such as atmospheric pressure chemical ionization (APCI), atmospheric pressure chemical photoionization (APPI), and negative ion chemical ionization (NICI). The GC-APCI-MS showed the lowest limits of detection from 30 to 300 nM for a broad range of fatty acids and a similar response for various fatty acids from a chain length of 10 to 20 carbon atoms. This allows the number of internal standards necessary for accurate quantification to be reduced. Moreover, the use of pentafluorobenzyl bromide allows the direct derivatization of free fatty acids making them accessible for GC–MS analysis without labor-intense sample pretreatment.

**Supplementary Information:**

The online version contains supplementary material available at 10.1007/s00216-022-04223-z.

## Introduction

For decades, lipids were considered a pure energy source, which have no role in biological systems. It was not until 1929 and 1930 that G.O. Burr and M.M. Burr showed that linoleic acid is essential for rats, as they developed deficiency symptoms on a linoleic acid-free diet [1, 2]. This demonstrated that some fatty acids (FAs) are essential and are playing an important role in biological systems [3]. Nowadays, the need for sensitive methods to determine FAs is increasing, as their analysis plays a major role in different areas such as system medicine or biology, as FAs are associated with inflammation effects like insulin resistance or nonalcoholic fatty liver disease [4, 5]. Additionally, their specific patterns and isotope distributions can be used to identify food fraud [6, 7], whereas, in the biological field, they are investigated to elucidate the composition of cell membranes, metabolic pathways, or evolutionary processes [8–10].

Although the majority of FAs are esterified to glycerol to form glycerophospholipids or mono-, di-, or triacylglycerides, a small proportion is also present as non-esterified or free fatty acids (FFAs) [11]. These are associated with various metabolic processes representing an analytically important target. For example, arachidonic acid is an important precursor for oxylipins which are able to activate the nuclear factor κB and can thereby lead to inflammatory effects [11, 12]. High levels of FFAs, when entering the liver, can lead to inflammation as well as insulin resistance and are also associated with diseases such as nonalcoholic fatty liver disease [5, 13]. When FFAs bind to a suitable receptor, they can serve as signaling molecules for the cell. FFA receptors are membrane molecules that are ubiquitously distributed in the human body. In particular, the FFA4 receptor not only is considered to be related to hormone excretion or glucose uptake but also plays an important role in the context of human cancer cells [14–17]. However, selective analysis of FFAs is still a challenge because they are often present at low concentrations in complex matrices, which usually contain other lipids as well as esterified FAs.

Gas chromatographic (GC) analysis of FAs has been reported since the 1950s [18, 19]. This analysis requires the derivatization of FAs to increase their volatility. The most widely used method for the quantification of FAs is their conversion into methylated derivatives [20–22]. However, it should be noted that the derivatization to form fatty acid methyl esters (FAMEs) may discriminate against individual substance classes depending on the derivatization strategy selected. For unsaturated FFAs, the derivatization efficiency of trimethylsulfonium hydroxide (TMSH) is less than 20% compared to the derivatization with NaOH and BF_3_, while both methods show no significant differences for saturated FFAs [20].

FAMEs as well as other derivatized FAs are usually detected by flame ionization detector (FID) or a mass spectrometer (MS). While FIDs are still important for the analysis of FAs, especially when analyzing the total fatty acid (TFA) profile [23–25], they do not provide mass spectral information, which results in a lack of specificity. Moreover, quantification is further complicated by the requirement for baseline separation of individual FAs. On the other hand, mass spectrometric methods mostly use electron ionization (EI), which allows qualitative as well as quantitative analysis of FAs [22]. This offers more information to identify FAs based on the EI fragmentation pattern as well as their retention time. However, fragmentation also leads to a compromise between sensitivity and selectivity for FAs analysis since the low abundance of the molecular ion (ca. < 25%) generally requires the monitoring of fragment ions at low *m/z* values. These fragment ions are generally common for most of FAs and could be strongly affected by ions coming from matrix interferences [26]. To sort out this issue, other softer ionization techniques such as chemical ionization (CI) or atmospheric pressure chemical ionization (APCI) have been used allowing the detection of the molecular ion [27, 28].

Derivatization with pentafluorobenzyl bromide (PFB) allows GC analysis of FAs followed by negative ion chemical ionization (NICI) leading to a clean MS-spectra with only the quasi-molecular ion [M-PFB]^−^. This derivatization was firstly used for organic acids [29] (1960s) but the major advantage for the analysis of FAs was not demonstrated until the 1980s through the use of NICI [30]. The electron capture properties of the PFB derivates are highly increased by the halogenated atoms, which enables an efficiencient formation of [M-H]^−^ ions with soft ionization techniques in negative mode [19], leading to significantly lower limits of detection (LODs) than FAMEs in positive mode when using a CI source [31]. Even though the PFB derivatization is not the most commonly used derivatization technique, it offers several advantages concerning specificity and selectivity, especially when FFAs or related compounds are analyzed in complex matrices as the PFB reagent is directly reacting with them [19, 32–34]. Therefore, no further sample preparation (e.g., SPE enrichment) is necessary.

While NICI technique is a well-known ion source that could promote [M-H]^−^ ions [35], other soft ionization techniques using APCI and atmospheric pressure photoionization (APPI) sources have also been used in the recent years for GC–MS, showing a great potential to ionize different families of compounds even in negative mode [27, 36, 37]. APCI ionization in negative mode is achieved by the corona discharge generated by a corona needle [38], while APPI uses a vacuum ultraviolet (VUV) lamp and requires an easily photoionizable substance (dopant), which later ionizes the analyte by gas-phase reactions [39]. These sources open new ionization mechanism that could help to improve the detection capabilities of GC–MS methods for FAs.

Here we have developed and compared GC–MS methods using APCI, APPI, and the standard NICI ion source for the selective and sensitive analysis of PFB derivatized FAs. The detection of FAs was carried out in selected ion monitoring (SIM) or *pseudo* selected reaction monitoring (SRM) mode. The methods include the *m/z* values or transitions for 75 different biologically relevant FAs (major FAs ranging from FA 8:0 up to FA 26:0 including several unsaturated FAs) to achieve their reliable determination in complex biological matrixes for an in-depth characterization of the FA profile and amount. To allow comparison between the different ionization techniques, the same GC parameters were chosen for all measurements and all methods were characterized according to the EMA guidelines for bioanalytical method validation. Finally, the applicability of the most suitable method was evaluated for the analysis of FFAs, in human plasma, human serum, and HepG2 cells.

## Materials and methods

### Chemicals and reagents

Caprylic acid (FA 8:0), myristic acid (FA 14:0), palmitic acid (FA 16:0), palmitoleic acid (FA 16:1 Δ9), stearic acid (FA 18:0), oleic acid (FA 18:1 Δ9), linoleic acid (FA 18:2 Δ9,12), and linolenic acid (FA 18:3 Δ9,12,15) were purchased from Sigma-Aldrich (Traufkirchen, Germany). Decanoic acid (FA 10:0), lauric acid (FA 12:0), arachidic acid (FA 20:0), arachidonic acid (FA 20:4 Δ5,8,11,14), eicosapentaenoic acid (FA 20:5 Δ5,8,11,14,17), docosanoic acid (FA 22:0), docosapentaenoic acid (FA 22:5 Δ7,10,13,16,19), docosahexaenoic acid (FA 22:6 Δ7,10,13,16,19), lignoceric acid (FA 24:0), and hexacosanoic (FA 26:0) acid as well as the stable isotope labeled internal standards ^2^H_2_-decanoic acid (^2^H_2_-FA 10:0), ^2^H_2_-pentadecylic acid (^2^H_2_-FA 15:0), ^2^H_4_-stearic acid (^2^H_4_-FA 18:0), ^2^H_8_-arachidonic acid (^2^H_8_-FA 20:4 Δ5,8,11,14), and ^2^H_4_-lignoceric acid (^2^H_4_-FA 24:0) were purchased from Cayman Chemical Company (Hamburg, Germany). 2,3,4,5,6-Pentafluorobenzyl bromide (for GC derivatization; ≥ 98.5%), acetone (≥ 99.9%), dietyl ether (≥ 99.0%), and N,N-diisopropylethylamine (≥ 99.5%) were purchased from Sigma-Aldrich (Traufkirchen, Germany). Dichloromethane (≥ 99.8%) was purchased from Thermo Fisher Scientific (Schwerte, Germany); toluene (HPLC grade), methyl tert-butyl ether (MTBE; LC–MS grade), tetrahydrofuran (≥ 99.9%), and acetic acid (LC–MS grade) were purchased from Merck (Darmstadt, Germany); and methanol (LC–MS grade) and benzene (≥ 99.0%) were purchased from Avantor (Darmstadt, Germany). Potassium hydroxide (pro analysi) was purchased from Bernd Kraft GmbH (Duisburg, Germany). Chlorobenzene (≥ 98%) was purchased from Fluka (Seelze, Germany). Ultrapure water with a resistivity of 18.2 M Ω/cm was desalted and filtered by a Sartorius Stedim water purification system (Sartorius, Goettingen, Germany). Human serum and human plasma were purchased by Sigma-Aldrich (Traufkirchen, Germany), and HepG2 cells were cultivated as described in Tötsch et al. [40].

### Lipid extraction

For FFA analysis, the samples were extracted according to Matyash [41] with some minor modifications. Briefly, 10 µL human plasma, serum, or 250,000 cells were spiked with 100 µL internal standard (each at a concentration of 2,000 nM), acidified with 5 µL acetic acid, and extracted by the addition of 300 µL methanol. The samples were homogenized in an ultrasonic bath, cooled by adding ice, for 5 min followed by the addition of 600 µL MTBE, and vortexed for 5 min. Afterwards, 300 µL water was added and the mixture was vortexed for 5 min. The upper phase was collected, and the aqueous phase was washed again with 300 µL MTBE. The combined MTBE phases were dried using a vacuum evaporator at 45 °C.

For the TFA analysis, samples were hydrolyzed prior to the described extraction. Therefore, 100 µL methanol and 60 µL potassium hydroxide (10 M) were added to the samples. The samples were homogenized in a cooled ultrasonic bath for 5 min and incubated at 60 °C for 30 min. The pH was increased by the addition of 70 µL of 50% acetic acid on ice, and the lipid extracts were dried using a vacuum evaporator at 45 °C.

The samples were directly derivatized and analyzed or stored at − 80 °C.

### Derivatization

The dried lipid extracts or standards were dissolved in 20 µL of 10% diisopropylethylamine in dichloromethane (1/9; *v/v*) and 20 µL of 10% 2,3,4,5,6‑pentafluorobenzylbromid in dichloromethane (1/9; *v/v*) was added. The samples were incubated at 50 °C for 1 h. After the incubation, the samples were dried using a vacuum evaporator at 45 °C and dissolved in 100 µL methanol, and analyzed.

### GC-APCI-MS and GC-APPI-MS analysis

The APCI measurements were performed using an Agilent 7890B GC equipped with a G4567A Autoinjector, and a DB-5 column (30 m × 250 μm × 0.25 μm), coupled with an APCI source (G312 — 69,100), and a 6495 triple Quad MS (Agilent Technologies, Waldbronn, Germany). 1 µL of the sample was injected into a split liner at 320 °C and operated with a split ratio of 1:10 and a septum purge flow of 1.5 mL/min. The column flow was set to 1.3 mL/min using helium as carrier gas. The temperature gradient was as followed: initial 100 °C, 15°/min linear to 160 °C, followed linear increase by 5°/min to 320 °C which was held for 5 min.

For the APPI measurements, the same GC–MS system with the same parameters for the gas chromatographic separation was used. However, the source was modified to perform photoionization. In detail, the APCI needle was removed and a capillary for the dopant was inserted into the source, which was connected to a syringe pump. The window of the APCI source chamber was removed and a krypton VUV lamp with the following specifications 10.6 eV, PKR 106, BH447, *λ* = 100–190 nm (Heraeus, Hanau, Germany) was installed in its place.

Both the APCI and the APPI were optimized as described below. The APCI measurements were performed with a corona current of 35 µA, a source gas flow of 11 L/min, a source gas temperature of 270 °C, a nitrogen auxiliary gas flow of 1 L/min, and a capillary voltage of 2000 V. The APPI measurements were performed with a VUV krypton lamp and toluene as reaction gas, a dopant flow of 20 µL/min, a source gas temperature of 200 °C, a capillary voltage of 2400 V, a source gas flow of 11 L/min, and the nitrogen auxiliary gas flow of 4 L/min. All measurements were performed in negative ionization and *pseudo* SRM mode with the *m/z* ratios of the [M-PFB]^−^ ions set as *m/z* for Q1 and Q3 with no fragmentation energy applied. The cycle time was 300 ms, which is sufficient to represent each peak with 12 data points. The *m/z* values of 75 different biologically relevant FAs covered by the method can be found in Table S1 in the electronic supplementary material (ESM).

### GC-NICI-MS analysis

The measurements with the NICI source were performed using a Shimadzu QP2010 Ultra from Shimadzu Deutschland (Duisburg, Germany) equipped with an AOC-20i Autoinjector from Shimadzu Deutschland, and a Zebron ZB-5MS column (30 m × 250 μm × 0.25 μm) from Phenomenex (Aschaffenburg, Germany) which has the same/similar stationary phase chemistry compared to the DB-5 column. The same GC parameters as for the Agilent GC-System described above were used. The NICI was operated with methane as the reaction gas and a source temperature of 280 °C. All measurements were performed in negative ionization and SIM mode with the *m/z* ratio of the [M-PFB]^−^ ions. The *m/z* values of 75 different biologically relevant FAs covered by the method can be found in Table S1 in the ESM.

### Data analysis

For instrument control, the Mass Hunter Workstation software GC/MS Acquisition (Version B.08.02) from Agilent was used for the APCI and the APPI measurements. The data analysis was done by Skyline-daily 21.0.9.118 from the University of Washington. Modde 12.1 (Sartorius Goettingen, Germany) was used for the design of experiment (DoE) to optimize APCI and APPI parameters. Data evaluation was performed using Microsoft Excel and GraphPad Prism (Version 5.02). The NICI measurements were analyzed using LabSolutions from Shimadzu Corporation.

## Results and discussion

### Chromatographic separation and detection

**Fig. 1:**
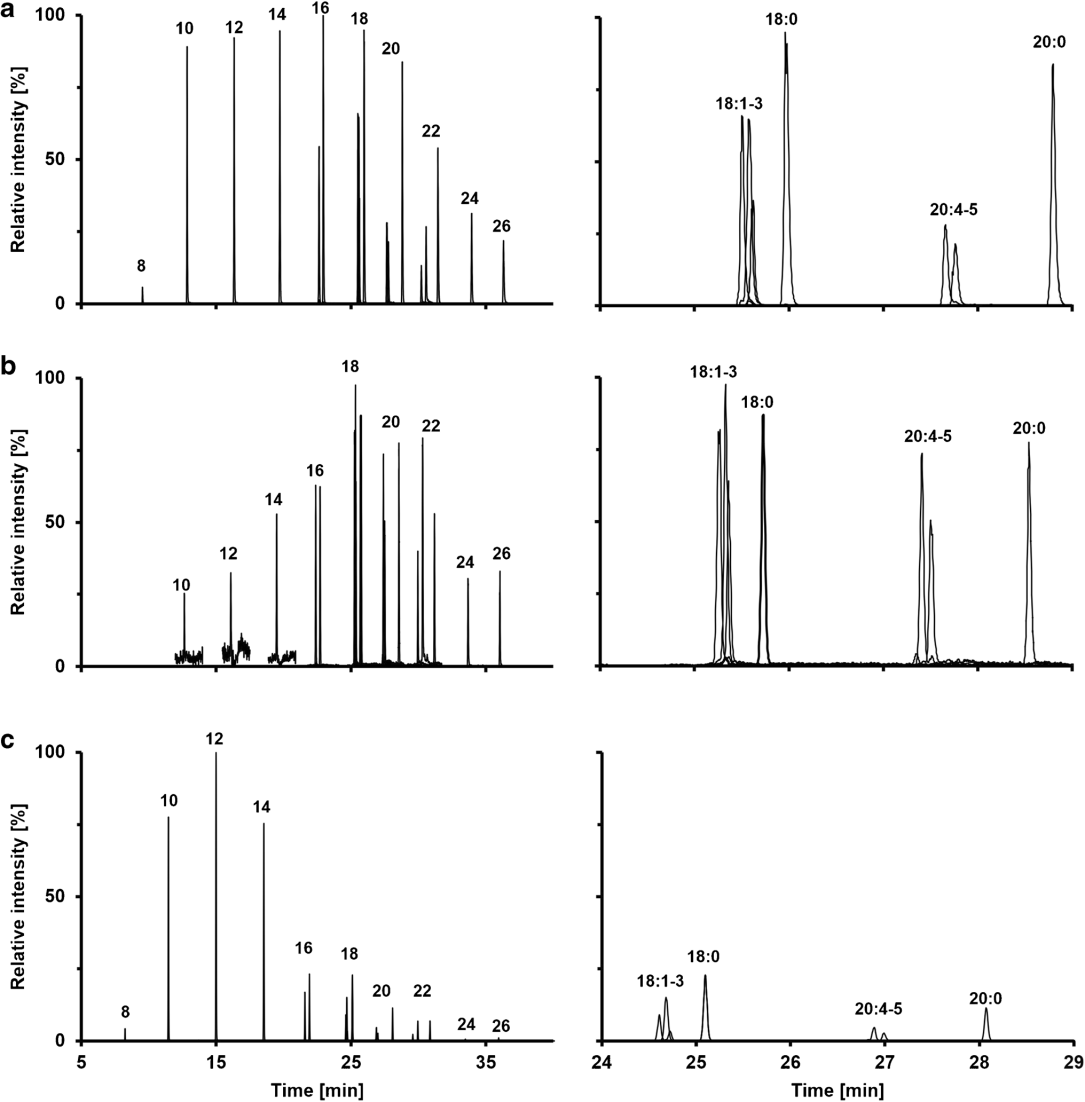
GC–MS analysis of a fatty acid standard of saturated and unsaturated fatty acids with a chain length from 8 to 26 carbon atoms and various degrees of double bonds all at a concentration of 30 µM. The same chromatographic conditions and injection volumes were used for all analyses. All saturated FAs are indicated by the number of carbon atoms in the figure. **a** Ionization was performed using APCI in *pseudo* SRM mode measuring the same *m/z* ratio in Q1 and Q3. **b** An APPI source was used for ionization. The detection was also performed in *pseudo* SRM mode. **c** Analysis was performed using a NICI source on a GC single quadrupole MS in SIM mode. While the ionization using APCI is yielding a uniform response for the FAs from C10 to C20, the APPI source showed higher ionization efficiency for longer chain FAs. In contrast, ionization by NICI shows a better performance for lower molecule FAs from C10 to C14

The gas chromatographic separation of FAMEs is usually carried out using polar columns (e.g., Wax or FFAP) [20, 42, 43]. However, the high boiling points of long-chain PFB derivatives and the different polarities of PFB derivatized FAs prevent their analysis using polar stationary phases. Thus, their separation is often performed using non-polar stationary phases such as DB-5 [19, 33]. The chromatographic separation of all ten saturated FAs, eight unsaturated FAs, and five internal standards using the APCI (A), APPI (B), and NICI source (C) is shown in Fig. 1. The applied method allows the separation of all major FAs based on the length of the carbon chain in 40 min.

As can be seen, most of the compounds are baseline separated, although some coelution was observed for the unsaturated FA 18:1 to 18:3 and FA 20:4 and 20:5 (Fig. 1). In addition, we were evaluating the separation of various FA 18:1 isomers after PFB derivatization. As can be seen in Figure S1, the separation of the n9 cis/trans isomers oleic acid and elaidic acid is also possible. Moreover, separation of different positional isomers (e.g., n7, n9, and n10) is also possible to a certain degree. However, as there is no fragmentation using the APCI, APPI, or NICI source, their individual determination can be achieved by monitoring the [M-PFB]^−^ ions which correspond to the *m/z* of the [M-H]^−^ ion. This is a major advantage compared to other GC methods using EI, leading to a more selective identification of FAs.

When comparing the results of the different ion sources, the APCI shows a similar ionization efficiency for saturated FAs with a chain length of 10 to 20 carbon atoms, leading to similar intensities (Fig. 1). This is a major advantage of APCI, since a similar response factor may reduce the number of external and internal standards. The home-made APPI source used was showing good performance for medium-chain FAs and long-chain FAs which might be related to the ionization mechanism of the dopant-assisted APPI. In contrast to APCI, where no dopant is required, the dopant-assisted APPI process highly depends on the dopant used, as their ionization energies could lead to a more or less efficient ionization of the analytes. Thus, depending on the gap of energy between the dopant and the analyte, the ionization efficiency of the target compounds could strongly vary using the same dopant. Unlike the other ion sources studied, the intensity of the unsaturated FAs is only slightly lower than the intensity of their respective saturated variants, and in some cases, the intensity of the unsaturated FAs is even exceeded. When FAs were analyzed with the NICI, there were greater differences in intensities compared with the other sources. In contrast to the APPI, the NICI source showed a very good performance for short- and medium-chain FAs while long-chain FAs showed lower sensitivities.

### Optimization of derivatization

Although derivatization could increase the volatility of the compounds, other properties, such as the stability of the analytes, or the ionization efficiency can also be changed. As mentioned before, although FAs are generally converted to FAMEs and subsequently analyzed by EI or FID [20–22], in this study, a PFB derivatization was used since it allows highly sensitive and selective ionization of the analytes in negative mode [19, 32, 33]. The PFB derivatization has been carefully optimized by considering different parameters such as volumes, incubation time, and the occurrence of background contamination that might be problematic when analyzing FAs in low concentrations.

**Fig. 2:**
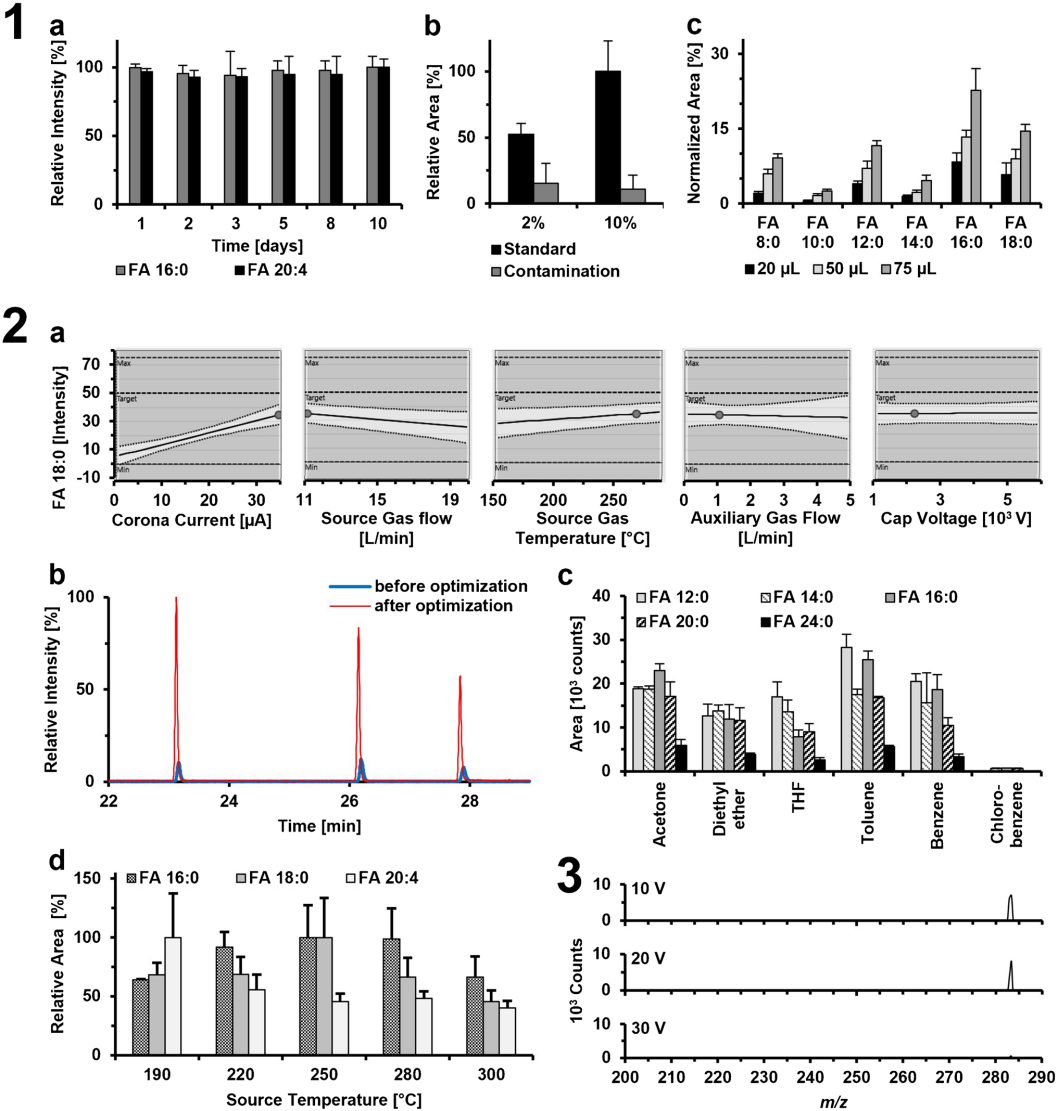
Optimization of FA analysis. **Part 1** Optimization of the PFB derivatization. 1a: Stability of the PFB derivates stored at room temperature was analyzed over 10 days. 1b: Influence of the concentration of the derivatization reagent (2% or 10%) with regard to background contamination is shown. The internal standard ^2^H_2_-FA 15:0 (1 µM) showed an increase in intensity while the background contamination (FA 8:0 to FA 18:1 summed up) showed no difference. 1c: Influence of the volume of the derivatization reagent on the background contamination for FAs 8:0, 10:0, 12:0, 14:0, 16:0, and 18:0. Higher volumes of derivatization reagent higher intensities for the depicted FAs were found while the intensity of the analyzed internal standard ^2^H_2_-FA 15:0 (1 µM) remains the same. **Part 2** Optimization of the different ion sources. 2a: The ionization conditions of APCI and APPI were optimized with a design of experiment (DoE) model, which is shown for the APCI optimization for FA 18:0. 2b: The increase in sensitivity of the APCI source is shown. The chromatogram shows FA 16:0, FA 18:0, and FA 20:4 with a concentration of 1 µM measured before and after method optimization showing a fivefold increase after optimization. 2c: Effect of different dopants on the intensities of FA 12:0, 14:0, 16:0, 20:0, and 24:0. 2d: Comparison of different source temperatures for the NICI source. **Part 3** Optimization of collision energies for the development of an SRM method. The mass spectrum shows a decrease in the intensity of the [M-PFB]^−^ ion for FA 18:0; however, no product ions were observed

First, the stability of PFB derivatized FAs was examined. For this, a mixture of FA 16:0 and FA 20:4 (10 µM each) was derivatized using the described PFB procedure and then analyzed by GC-APCI-QqQ-MS. FA 18:0 was used to normalize the area of the detected peaks. The samples were stored at room temperature and analyzed on different days after preparation. As shown in Fig. 2 part 1a, no decrease in the relative amount of the derivatized FAs could be detected within a period of 10 days, which meant a great advantage in terms of sample handling, especially for highly unsaturated FAs such as FA 20:4.

Regarding the PFB derivatization, it should proceed completely which is consistent with maximizing the peak intensity of the analytes while the intensity of background contamination should be minimized. Contamination is a well-known problem in FA analysis, mainly FA 16:0 and FA 18:0, but in smaller quantities, also short- and medium-chain FAs are known to be contaminants, resulting in an increase of LOD values for FAs with high background contaminations [24, 32, 44]. Because of this, all chemicals and equipment used should be carefully monitored. Nevertheless, due to the ubiquitous occurrence of FAs, it was not possible to eliminate the background completely. Therefore, the concentration and the volume of the derivatization reagent were optimized as well. For this purpose, ^2^H_2_-FA 15:0 (1 µM) was derivatized and subsequently, the intensity of the ^2^H_2_-FA 15:0 was compared with the intensities of known contaminations (FA 8:0 to FA 18:1 summed up). It was found that a lower amount of FA was derivatized when using derivatization reagent containing only 2% PFB/diisopropylethylamine (DiPEA) compared to 10%, while the background signals had about the same height in both cases (Fig. 2 part 1b). Therefore, to ensure complete derivatization, the concentration of the derivatizing agent was increased to 10%. Different volumes of 10% derivatization reagent were then analyzed. There were no significant differences in the signal of the analyte, but the contamination signals increased with increasing volume (Fig. 2 part 1c). To ensure quantitative derivatization and a minimum background, 20 µL of each reagent was used.

### Optimization of ionization and detection parameters

To achieve sensitive detection of PFB derivatized FAs, the source parameters were carefully optimized. Additionally, the most suitable transitions and collision energies were chosen to achieve a selective determination of the analytes.

For the GC-APCI coupling, corona current, source gas flow, source gas temperature, nitrogen auxiliary gas flow, and capillary voltage were optimized by means of a DoE. A linear process model was used with 61 experiments in total, which is shown in Fig. 2 part 2a. The corona current, source gas flow, and gas temperature were found the most important factors for the ionization of PFB derivatized FAs while the impact of other parameters was neglectable. These optimized parameters were chosen and subsequently tested. Figure 2 part 2b shows two chromatograms before and after the optimization, leading to a response improvement of fivefold.

For the APPI source, different dopants were tested (acetone, diethyl ether, tetrahydrofuran, toluene, benzene, and chlorobenzene). As shown in Fig. 2 part 2c, toluene was found to be the most suitable for the ionization of PFB derivatized FAs. Subsequently, the dopant flow, source gas temperature, capillary voltage, source gas flow, and nitrogen auxiliary gas flow were optimized using a DoE approach. The DoE model was not completely accurate in predicting the best parameters because of the minimal differences between the individual parameters. Therefore, the values were individually checked and adjusted afterwards.

Following the source optimization, different collision cell voltages were tested using the APCI source (Fig. 2 part 3). The aim was to improve the selectivity of the method by implementing a selective reaction monitoring (SRM) method. To do that, product ion scans from the [M-PFB]^−^ precursor ion were carried out from *m/z* 50 to 180 for FA 10:0, from *m/z* 50 to 290 for FA 18:0, and from *m/z* 50 to 380 for FA 24:0 (6 µM each). As expected, although it was shown that the [M-PFB]^−^ ion intensities were reduced with increasing collision energy, no abundant product ions were found. Therefore, *pseudo* SRM method was proposed by monitoring [M-PFB]^−^ to [M-PFB]^−^ transitions. Table 1 shows all *m/z* ratios, retention times, and peak width of the analyzed FAs. However, the final list of *m/z* ratios that were programmed contained the *m/z* ratios of a total of 75 FAs to also be able to detect these FAs in biological samples (Table S1). This allows the analysis of a large number of FAs without increasing the LOD values due to the shorter dwell times per FA.

**Table 1:** Chromatographic performance of the different GC–MS analysis. The *m/z* ratios correspond to the *pseudo* SRM and SIM analysis. The internal standard used for quantification, retention time (*t*_R_), and the full peak width and the full width at half maximum including the standard deviation (*n* = 3)

FA	*m/z*	Internal standard			*t*_R_ [min]			Peak width [s]					
								Full width*			FWHM*		
		APCI	APPI	NICI	APCI	APPI	NICI	APCI	APPI	NICI	APCI	APPI	NICI
**8:0**	143.2	^2^H_2_-FA 10:0	^2^H_4_-FA 18:0	^2^H_2_-FA 10:0	9.54 ± 0.01	n.a	8.25 ± 0.01	0.12 ± 0.00	n.a	0.09 ± 0.01	0.04 ± 0.00	n.a	0.03 ± 0.00
**10:0**	171.2	^2^H_2_-FA 10:0	^2^H_4_-FA 18:0	^2^H_2_-FA 10:0	12.84 ± 0.01	n.a	11.46 ± 0.02	0.13 ± 0.01	n.a	0.10 ± 0.01	0.04 ± 0.01	n.a	0.04 ± 0.00
**12:0**	199.3	^2^H_2_-FA 15:0	^2^H_4_-FA 18:0	^2^H_2_-FA 15:0	16.33 ± 0.01	16.09 ± 0.01	14.99 ± 0.01	0.16 ± 0.01	0.09 ± 0.01	0.13 ± 0.00	0.05 ± 0.00	0.04 ± 0.00	0.04 ± 0.01
**14:0**	227.4	^2^H_2_-FA 15:0	^2^H_4_-FA 18:0	^2^H_2_-FA 15:0	19.72 ± 0.01	19.50 ± 0.00	18.53 ± 0.01	0.16 ± 0.02	0.10 ± 0.00	0.11 ± 0.01	0.05 ± 0.00	0.06 ± 0.00	0.04 ± 0.00
**16:0**	255.5	^2^H_4_-FA 18:0	^2^H_4_-FA 18:0	^2^H_4_-FA 18:0	22.95 ± 0.01	22.71 ± 0.01	21.90 ± 0.02	0.15 ± 0.01	0.11 ± 0.02	0.12 ± 0.01	0.07 ± 0.01	0.05 ± 0.00	0.04 ± 0.00
**16:1**	253.5	^2^H_4_-FA 18:0	^2^H_4_-FA 18:0	^2^H_4_-FA 18:0	22.62 ± 0.00	22.39 ± 0.01	21.57 ± 0.02	0.14 ± 0.01	0.10 ± 0.02	0.10 ± 0.01	0.05 ± 0.02	0.05 ± 0.00	0.04 ± 0.00
**18:0**	283.5	^2^H_4_-FA 18:0	^2^H_4_-FA 18:0	^2^H_4_-FA 18:0	25.97 ± 0.01	25.72 ± 0.01	25.08 ± 0.01	0.16 ± 0.00	0.10 ± 0.02	0.13 ± 0.03	0.06 ± 0.01	0.05 ± 0.01	0.04 ± 0.01
**18:1**	281.5	^2^H_4_-FA 18:0	^2^H_4_-FA 18:0	^2^H_4_-FA 18:0	25.57 ± 0.01	25.34 ± 0.01	24.67 ± 0.01	0.16 ± 0.02	0.09 ± 0.03	0.12 ± 0.01	0.05 ± 0.01	0.04 ± 0.00	0.05 ± 0.01
**18:2**	279.5	^2^H_4_-FA 18:0	^2^H_4_-FA 18:0	^2^H_4_-FA 18:0	25.49 ± 0.01	25.26 ± 0.01	24.60 ± 0.02	0.14 ± 0.02	0.09 ± 0.01	0.10 ± 0.01	0.05 ± 0.01	0.04 ± 0.01	0.04 ± 0.00
**18:3**	277.5	^2^H_4_-FA 18:0	^2^H_4_-FA 18:0	^2^H_4_-FA 18:0	25.60 ± 0.02	25.37 ± 0.00	24.71 ± 0.02	0.11 ± 0.01	0.13 ± 0.00	0.10 ± 0.01	0.05 ± 0.02	0.05 ± 0.00	0.05 ± 0.00
**20:0**	311.5	^2^H_8_-FA 20:4	^2^H_8_-FA 20:4	^2^H_8_-FA 20:4	28.80 ± 0.01	28.54 ± 0.01	28.07 ± 0.02	0.16 ± 0.02	0.09 ± 0.02	0.10 ± 0.02	0.06 ± 0.01	0.05 ± 0.01	0.05 ± 0.00
**20:4**	303.5	^2^H_8_-FA 20:4	^2^H_8_-FA 20:4	^2^H_8_-FA 20:4	27.67 ± 0.01	27.41 ± 0.00	26.87 ± 0.02	0.13 ± 0.01	0.10 ± 0.00	0.11 ± 0.01	0.07 ± 0.02	0.05 ± 0.00	0.05 ± 0.00
**20:5**	301.5	^2^H_8_-FA 20:4	^2^H_8_-FA 20:4	^2^H_8_-FA 20:4	27.76 ± 0.01	27.50 ± 0.00	26.99 ± 0.02	0.12 ± 0.02	0.15 ± 0.00	0.11 ± 0.01	0.04 ± 0.02	0.06 ± 0.00	0.05 ± 0.00
**22:0**	339.6	^2^H_4_-FA 24:0	^2^H_4_-FA 24:0	^2^H_4_-FA 24:0	31.45 ± 0.02	31.19 ± 0.01	30.85 ± 0.02	0.14 ± 0.02	0.12 ± 0.01	0.11 ± 0.01	0.05 ± 0.02	0.05 ± 0.01	0.04 ± 0.00
**22:5**	329.5	^2^H_4_-FA 24:0	^2^H_4_-FA 24:0	^2^H_4_-FA 24:0	30.57 ± 0.02	30.32 ± 0.00	29.95 ± 0.01	0.16 ± 0.02	0.16 ± 0.00	0.11 ± 0.02	0.06 ± 0.01	0.05 ± 0.00	0.05 ± 0.01
**22:6**	327.5	^2^H_4_-FA 24:0	^2^H_4_-FA 24:0	^2^H_4_-FA 24:0	30.20 ± 0.01	29.95 ± 0.00	29.56 ± 0.02	0.14 ± 0.02	0.12 ± 0.00	0.13 ± 0.03	0.06 ± 0.01	0.05 ± 0.00	0.05 ± 0.01
**24:0**	367.7	^2^H_4_-FA 24:0	^2^H_4_-FA 24:0	^2^H_4_-FA 24:0	33.96 ± 0.01	33.67 ± 0.01	33.46 ± 0.02	0.18 ± 0.03	0.11 ± 0.01	0.11 ± 0.01	0.07 ± 0.02	0.06 ± 0.01	0.05 ± 0.00
**26:0**	395.7	^2^H_4_-FA 24:0	^2^H_4_-FA 24:0	^2^H_4_-FA 24:0	36.29 ± 0.01	36.03 ± 0.02	35.92 ± 0.01	0.30 ± 0.09	0.12 ± 0.06	0.11 ± 0.02	0.11 ± 0.04	0.05 ± 0.00	0.05 ± 0.01

^*^Full width and Full width half maximum (FWHM) at LOQ of the corresponding FA

The NICI source is mainly optimized during the tune of the instrument which includes the ionization voltage, ion optics, and detector-specific parameters. However, the temperature plays an important role in the ionization process as well as the robustness of the method. Therefore, the impact of different temperatures was investigated on the response of different FAs as well as human plasma, human serum, and cell samples. As can be seen from Fig. 2 part 2d, 250 °C is providing the highest response for the investigated FAs. However, a rapid decrease in the response was observed when analyzing samples at this temperature probably due to contamination of the source chamber. Therefore, a higher temperature of 280 °C was used for the analysis [19]. Here no decrease in the response was observed when analyzing biological samples with a high matrix load which increases the robustness.

### Method characterization

In order to characterize and compare the method performance, the LOD (signal-to-noise ratio (S/N) of ≥ 3) and the lower limit of quantification (LLOQ) (S/N of ≥ 5 and accuracy of ± 20%) were determined according to the EMA guidelines for bioanalytical method validation [45].

The LODs for the GC-APCI-MS method were between 30 and 300 nM (3 to 30 fmol or 0.4 to 8 pg on column), for the APPI generally between 100 and 3,000 nM (10 to 300 fmol or 2 to 90 pg on column), and for the NICI between 10 and 1,000 nM (1 to 100 fmol on column or 0.2 to 100 pg on column) (Table 2). However, for both the APPI and the NICI source, rather high LODs were observed for some short-chain FAs. While the FA 8:0 was not detectable with the APPI, the other two shorter chain FAs (FA 10:0 and FA 12:0) showed rather high LODs of 30,000 and 20,000 nM, respectively. For the NICI source, also the FA 8:0 was rather problematic to detect which results in a LOD of 6,000 nM.

**Table 2:** Comparison of the validation data for all investigated ion sources and analyzed FAs. Method characterization was carried out according to the EMA guidelines [45]. The LOD is calculated with an S/N of ≥ 3, the LLOQ with an S/N of ≥ 5, and an accuracy of ± 20% and the LLOQ as the highest analyzed concentration with an accuracy of ± 20%

FA	LOD [nM]			LOD on-column [fmol]			LOD on-column [pg]			Calibration range [nM]						Accuracy [%]						*R*^2^*		
										LLOQ			ULOQ			LLOQ			ULOQ					
	APCI	APPI	NICI	APCI	APPI	NICI	APCI	APPI	NICI	APCI	APPI	NICI	APCI	APPI	NICI	APCI	APPI	NICI	APCI	APPI	NICI	APCI	APPI	NICI
**8:0**	30	n.a	6,000	2.7	n.a	550	0.39	n.a	79	100	n.a	10,000	1,000	n.a	30,000	99	n.a	107	96	n.a	139	0.996	n.a	0.812
**10:0**	100	30,000	100	9.1	2,700	9.1	1.6	470	1.6	300	n.a	300	20,000	n.a	30,000	96	n.a	105	87	n.a	93	0.981	n.a	0.984
**12:0**	100	20,000	30	9.1	1,800	2.7	1.8	360	0.54	300	n.a	100	30,000	n.a	30,000	103	n.a	101	103	n.a	114	0.992	n.a	0.978
**14:0**	30	300	10	2.7	27	0.91	0.62	6.2	0.21	100	3,000	30	30,000	30,000	30,000	98	104	90	100	107	104	0.996	0.983	0.978
**16:0**	300	100	1,000	27	9.1	91	6.9	2.3	23	1,000	300	3,000	30,000	20,000	20,000	105	85	106	89	90	108	0.970	0.915	0.977
**16:1**	30	1,000	100	2.7	91	9.1	0.69	23	2.3	100	1,000	300	30,000	30,000	30,000	106	96	98	93	97	87	0.974	0.979	0.993
**18:0**	300	100	300	27	9.1	27	7.8	2.6	7.8	1,000	300	1,000	30,000	30,000	30,000	102	86	99	101	85	103	0.997	0.926	0.982
**18:1**	100	300	300	9.1	27	27	2.6	7.7	7.7	300	300	1,000	30,000	30,000	20,000	102	103	101	91	92	108	0.985	0.972	0.994
**18:2**	30	1,000	100	2.7	91	9.1	0.78	26	2.6	100	1,000	300	30,000	30,000	20,000	106	95	99	94	96	101	0.974	0.973	0.997
**18:3**	30	3,000	300	2.7	270	27	0.76	76	7.6	100	3,000	1,000	30,000	30,000	20,000	103	94	100	99	101	105	0.991	0.962	0.991
**20:0**	30	1,000	100	2.7	91	9.1	0.84	28	2.8	100	1,000	300	30,000	30,000	30,000	94	112	100	105	115	115	0.980	0.944	0.987
**20:4**	30	3,000	300	2.7	270	27	0.83	83	8.3	100	3,000	1,000	30,000	30,000	30,000	98	101	100	92	106	93	0.996	0.989	0.996
**20:5**	100	3,000	300	9.1	270	27	2.8	83	8.3	100	3,000	1,000	30,000	30,000	30,000	105	99	101	114	103	98	0.984	0.983	0.993
**22:0**	30	1,000	100	2.7	91	9.1	0.92	31	3.1	100	1,000	300	30,000	30,000	10,000	106	98	97	92	111	102	0.995	0.979	0.993
**22:5**	100	3,000	100	9.1	270	9.1	3.0	90	3.0	300	3,000	300	30,000	20,000	20,000	102	107	105	94	114	115	0.990	0.964	0.984
**22:6**	100	3,000	1,000	9.1	270	91	3.0	90	30	300	3,000	3,000	20,000	30,000	30,000	100	104	100	99	115	102	0.999	0.975	0.998
**24:0**	100	1,000	3,000	9.1	91	270	3.4	34	100	300	6,000	6,000	30,000	30,000	30,000	98	108	103	92	103	93	0.996	0.960	0.973
**26:0**	100	1,000	300	9.1	91	27	3.6	36	11	300	10,000	1,000	30,000	30,000	10,000	97	103	97	96	108	91	0.993	0.966	0.985
**Median**	**100**	**1000**	**300**	**9.1**	**91**	**27**	**1.7**	**34**	**7.7**	**200**	**3,000**	**1,000**	**30,000**	**30,000**	**30,000**	**102**	**101**	**100**	**95**	**103**	**103**	**0.992**	**0.972**	**0.986**

^*^R^2^ of an 1/*x*^2^ weighted linear regression; *n* = 3

These LODs are comparable to those GC methods reported in the literature which are in the range of fmols on column, depending on the FA. For instance, Kish-Trier et al. were using a GC-NICI-MS method and are reporting LODs from 5 to 500 fmol on column for FAs from FA 12:0 to FA 24:1, while Schött et al. were using a GC-EI-MS and are reporting LODs from 0.4 to 110 fmol on column for FAs including FA 10:0 to FA 29:0 [46, 47]. Moreover, LC–MS has been increasingly used for the analysis of FAs in recent years due to the spread of mass spectrometry. Unlike GC, it offers the possibility of measuring FFA without prior derivatization [44]. The often used electrospray ionization coupled with a mass spectrometer (ESI–MS) provides the advantage that FAs can be ionized without fragmentation, which facilitates the identification of the FAs [44, 48, 49]. However, a comparison is rather difficult due to the of use split injection in GC. A recently published method showed slightly lower LODs using LC–MS in the range from 5 to 100 nM for a wide range of investigated FAs while the on column LODs (50 to 1000 fmol on column) were slightly higher compared to GC-APCI-MS method reported here [44, 50].

### Robustness

**Fig. 3:**
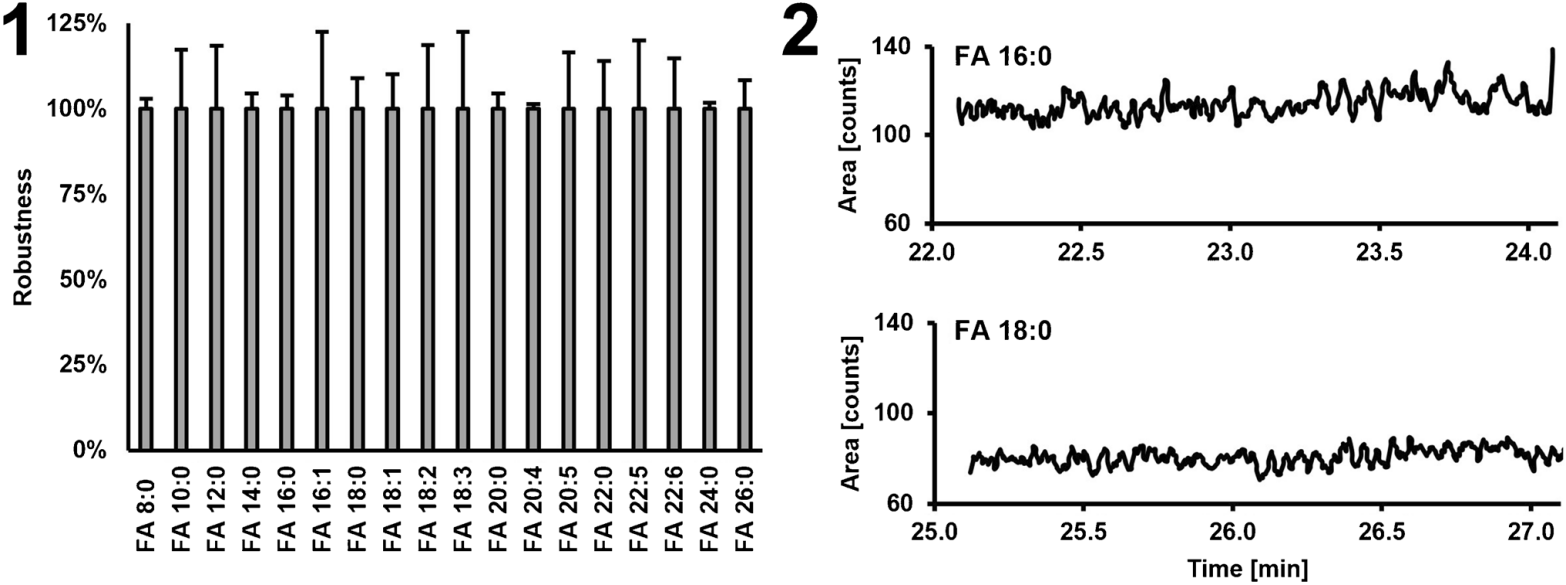
**Part 1** Standards measured at the respective LLOQ were analyzed by GC-APCI-MS on three different days and the corresponding variance was calculated for each FA. The variance for all FAs was below 20%, except FA 16:1 and FA 18:3 (23% each). **Part 2** To determine the carry-over effect for the GC-APCI-MS, a standard with a concentration of 30 µM was injected followed by the analysis of a blank sample of pure methanol. As shown for FA 16:0 and FA 18:0, no peak is visible resulting in a low carry-over (below 20% of the LLOQ)

For the day-to-day variance of the method, FA standard mixtures were derivatized and analyzed on 3 subsequent days using the GC-APCI-MS because of the most promising results obtained. As shown in Fig. 3 part 1, all variances measured at the respective LLOQ were below 20%, except FA 16:1 and FA 18:3 which were at 23% each. Taking into account the standard deviations of APCI ion sources described in the literature with approximately 10 to 60%, this could be attributed primarily to the ion source [51]. In combination with the high stability of the PFB derivatives (Fig. 2 part 1a), this enables the measurement of longer sample sequences. To study carry-over effects, highly concentrated standards and samples were analyzed followed by the injection of pure methanol (underivatized). It was examined if there is either a carry-over from one to another sample, e.g., due to the syringes. As shown in Fig. 3 part 2 using the example of FA 16:0 and FA 18:0, no carry-over could be detected, thus fulfilling the requirements of the EMA guideline (carry-over may not exceed 20% of the LLOQ).

### Analysis of fatty acids in biological samples

After validation and comparison of all methods, the most promising method (GC-APCI-MS/MS) was tested to determine FFAs in clinically and biologically relevant samples such as plasma, serum, and HepG2 cells. The extraction was carried out as described by Matyash et al. [41] using MTBE. Then, the extracts were derivatized using PFB as previously mentioned and the samples were measured by GC-APCI-MS. As can be seen from Fig. S2 in the ESM, the recovery of the internal standards (except for FA 10:0 ^2^H_2_) was around 95%.

**Fig. 4:**
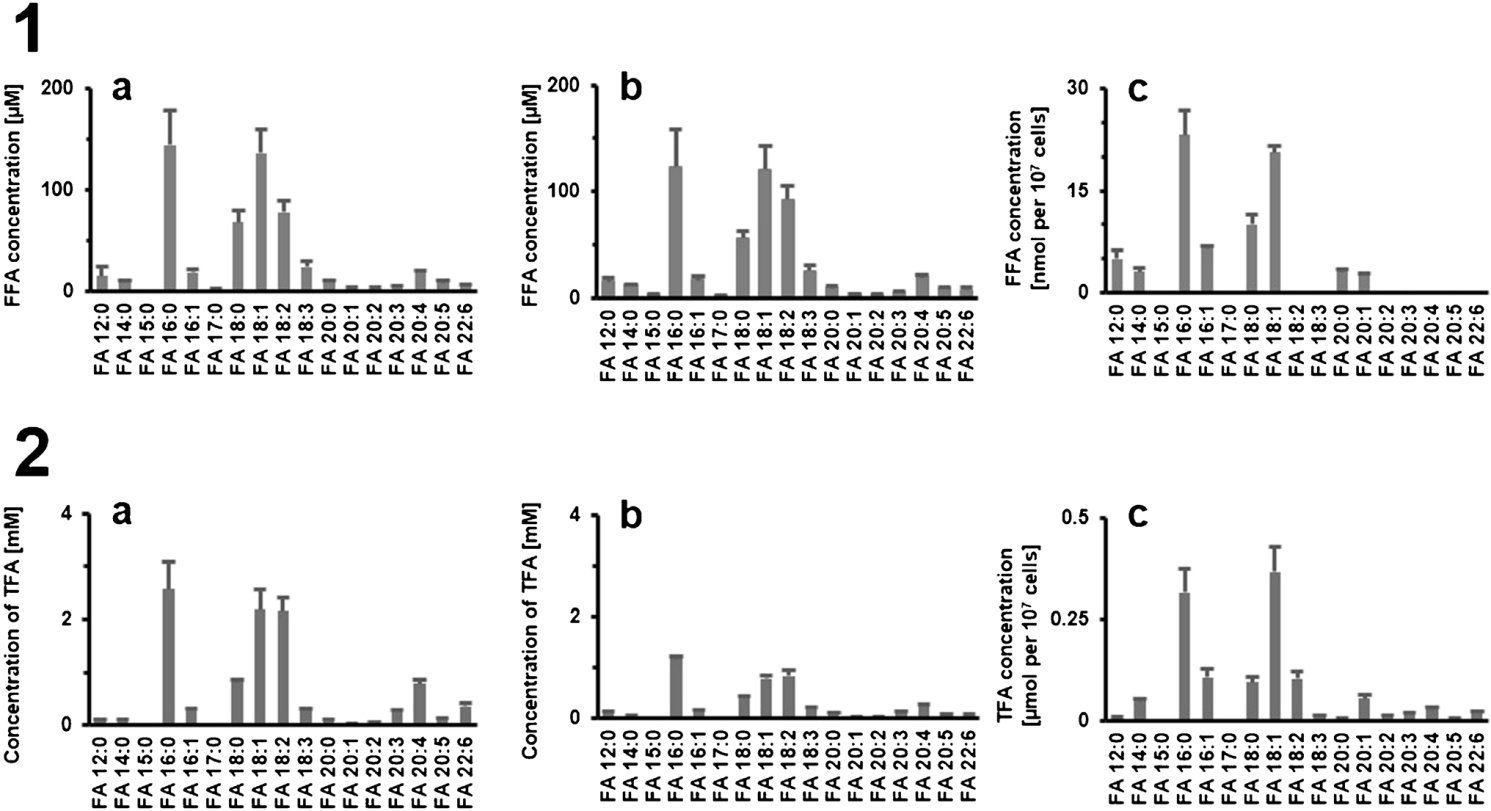
Quantification of free FAs and total FAs in plasma, serum, and cells by GC-APCI-MS. The samples (a: plasma, b: serum, and c: HepG2 cells; *n* = 5) were extracted according to Matyash and derivatized with PFB (**Part 1**). For TFAs, the samples were hydrolyzed using KOH prior to extraction and derivatization (**Part 2**). Analysis was carried out using the described GC-APCI-QqQ method in *pseudo* SRM mode. Concentrations of the FFA and TFA were determined using authentic standards and stable isotope labeled internal standards

As Fig. 4 shows, the highest concentrations of FFAs were found for FA 16:0, FA 18:0, FA 18:1, and FA 18:2. In addition, FA 12:0, FA 14:0, FA 20:0, and several unsaturated FFAs with 20 carbons were found in all samples. Moreover, small amounts of FA 22:6 were detected in plasma and serum, which were not detected in HepG2 cells. The FFA content determined in plasma is in good agreement with the literature. The concentrations of FA 18:1 were 136 ± 23 µM compared to 110 µM and for FA 18:2 78 ± 11 µM compared to 44 µM previously reported [52]. However, differences were found for FA 16:0 and FA 18:0 in the human plasma (144 ± 34 µM and 68 ± 12 µM) compared to the concentrations reported by Bowden et al. (43 µM and 15 µM, respectively) [52]. However, as described above, these two fatty acids are frequent contaminants, resulting in high relative standard deviations. In addition, some FAs were found in low concentrations, which were previously described only as TFA, such as FA 12:0, FA 14:0, or FA 20:5. The content of most FFAs quantified in serum is lower than the values described in the literature. The determined concentrations for FA 16:0 are 123 ± 36 µM compared to 234 µM, for FA 18:0 56 ± 6 µM compared to 222 µM, and for FA 18:2 93 ± 13 µM compared to 182 µM previously reported by Zhao et al. [53]. For FA 18:1, the determined concentration of 121 ± 22 µM is in good agreement with 131 µM reported by Zhao et al. [53].

As only free fatty acids can be detected by PFB derivatization, hydrolysis must be carried out prior to derivatization in order to determine the TFA content. In this process, fatty acids that are bound in complex lipids such as triacylglycerides or glycerophospholipids are saponified and can then be derivatized using PFB. All other steps were carried out the same way as for the determination of FFAs. TFA levels determined in plasma were also in agreement with literature values. For example, the concentrations of FA 16:0 determined by the described GC-APCI-MS method were 2579 ± 504 µM compared to 2360 µM reported by Kish-Trier et al. [47] and 2470 µM reported by Cruz-Hernandez et al. [54]. For FA 18:1, we found 2185 ± 378 µM compared to 1610 µM reported by Kish-Trier et al. [47] and 2320 µM reported by Cruz-Hernandez et al. [54], and for FA 18:2 2161 ± 245 µM compared to 2840 µM reported by Kish-Trier et al. [47] and 1690 µM reported by Cruz-Hernandez et al. [54]. As with FFA concentration, the TFA concentration in the serum samples was lower than the values reported in the literature. For instance, the concentrations determined by GC-APCI-MS were for FA 16:0 1170 ± 61 µM compared to 2340 to 2680 µM [42, 43, 55]. For FA 18:1, we found 786 ± 54 µM in the serum sample compared to 1770 to 2640 µM, and for FA 18:2 830 ± 121 µM compared to 3430 to 3850 µM reported in the literature [42, 43, 55].

It should be noted that the used plasma, as well as serum, were from a different supplier compared to those reported in the literature. Also, it has to be taken into consideration that no standardized plasma or serum was used and some of the FAs mentioned are having high deviations within and between the different publications as well. This can be explained by large biological variations [46, 56] and of course, some differences in the analytical methods used. Overall, the quantified FA concentrations are therefore in good agreement with concentrations reported in the literature.

## Conclusion

For the analysis of non-esterified FAs, a method based on GC-APCI-MS using PFB as derivatization agent was developed. Moreover, different ion sources such as APCI, APPI, and NICI were compared. The GC-APCI-MS showed the lowest LODs (from 30 to 300 nM) and LLOQs (from 100 to 1000 nM) for a broad range of FAs and a similar response for several FAs (FA 10:0 to 18:0). As APCI is less prone to matrix interferences (Fig. S3) such as ion suppression or ion enhancement and because of the rather similar response for many FAs, it is possible to reduce the number of internal standards necessary for accurate quantification. Moreover, the use of PFB allows the direct derivatization of FFAs making them accessible for GC–MS analysis without labor-intense sample pretreatment. In addition, the PFB derivates showed high stability allowing the measurement over several days without the risk of FA degradation. The application of the method to analyze plasma, serum, and HepG2 cells showed comparable levels with other previous reports [42, 43, 47, 52–55] and demonstrates the good performance of the method for the analysis of FFAs in the presence of other lipids (e.g., phospholipids, sphingolipids, and triacylglycerols) without the need for any sample clean-up.

## Supplementary Information

Below is the link to the electronic supplementary material.

Supplementary file1 (DOCX 77 KB)
