# Supplementary material for: Potential of atmospheric pressure ionization sources for the analysis of free fatty acids in clinical and biological samples by gas chromatography-mass spectrometry
Source: Anal Bioanal Chem. 2022 Jul 18;414(22):6621–34. doi: 10.1007/s00216-022-04223-z (PMC9411222; doi:10.1007/s00216-022-04223-z)
Supplement: Supplementary file 1 — Supplementary file1 (DOCX 77 KB) [file 216_2022_4223_MOESM1_ESM.docx]

**Potential of atmospheric pressure ionization sources for the analysis of free fatty acids in clinical and biological samples by gas chromatography-mass spectrometry**

Paul E. Görs, Pia Wittenhofer, Juan F. Ayala-Cabrera, Sven W. Meckelmann

Applied Analytical Chemistry, University of Duisburg-Essen, Universitätsstrasse 5, 45141 Essen, Germany

Table S1: m/z values of all analyzed fatty acids. All standards that were used as standards are printed bold.

| **Compound** | **m/z** |  | **Compound** | **m/z** |
| --- | --- | --- | --- | --- |
| FA 6:0 | 115.2 |  | FA 21:0 | 325.5 |
| FA 7:0 | 129.2 |  | FA 21:1 | 323.5 |
| **FA 8:0** | **143.2** |  | FA 21:2 | 321.5 |
| FA 9:0 | 157.2 |  | FA 21:3 | 319.5 |
| **FA 10:0** | **171.2** |  | **FA 22:0** | **339.6** |
| **^2^H_2_-FA 10:0** | **173.3** |  | FA 22:1 | 337.6 |
| FA 10:1 | 169.2 |  | FA 22:2 | 335.6 |
| FA 11:0 | 185.3 |  | FA 22:3 | 333.6 |
| FA 11:1 | 183.3 |  | FA 22:4 | 331.6 |
| **FA 12:0** | **199.3** |  | **FA 22:5** | **329.5** |
| FA 12:1 | 197.3 |  | **FA 22:6** | **327.5** |
| FA 13:0 | 213.4 |  | FA 23:0 | 353.6 |
| FA 13:1 | 211.4 |  | FA 23:1 | 351.6 |
| **FA 14:0** | **227.4** |  | FA 23:2 | 349.6 |
| FA 14:1 | 225.4 |  | FA 23:3 | 347.6 |
| FA 15:0 | 241.4 |  | FA 23:4 | 345.6 |
| **^2^H_2_-FA 15:0** | **243.4** |  | FA 23:5 | 343.6 |
| FA 15:1 | 239.4 |  | FA 23:6 | 341.6 |
| **FA 16:0** | **255.5** |  | **FA 24:0** | **367.7** |
| **FA 16:1** | **253.5** |  | **^2^H_4_-FA 24:0** | **371.7** |
| FA 16:2 | 251.5 |  | FA 24:1 | 365.7 |
| FA 17:0 | 269.5 |  | FA 24:2 | 363.7 |
| FA 17:1 | 267.5 |  | FA 24:3 | 361.7 |
| **FA 18:0** | **283.5** |  | FA 24:4 | 359.7 |
| **^2^H_4_-FA 18:0** | **287.5** |  | FA 24:5 | 357.7 |
| **FA 18:1** | **281.5** |  | FA 24:6 | 355.7 |
| **FA 18:2** | **279.5** |  | FA 25:0 | 381.7 |
| **FA 18:3** | **277.5** |  | FA 25:1 | 379.7 |
| FA 18:4 | 275.5 |  | FA 25:2 | 377.7 |
| FA 19:0 | 297.5 |  | **FA 26:0** | **395.7** |
| FA 19:1 | 295.5 |  | FA 26:1 | 393.7 |
| FA 19:2 | 293.5 |  | FA 26:2 | 391.7 |
| **FA 20:0** | **311.5** |  | FA 26:3 | 389.7 |
| FA 20:1 | 309.5 |  | FA 26:4 | 387.7 |
| FA 20:2 | 307.5 |  | FA 26:5 | 385.7 |
| FA 20:3 | 305.5 |  | FA 26:6 | 383.7 |
| **FA 20:4** | **303.5** |  |  |  |
| **^2^H_8_-FA 20:4** | **311.5** |  |  |  |
| **FA 20:5** | **301.5** |  |  |  |


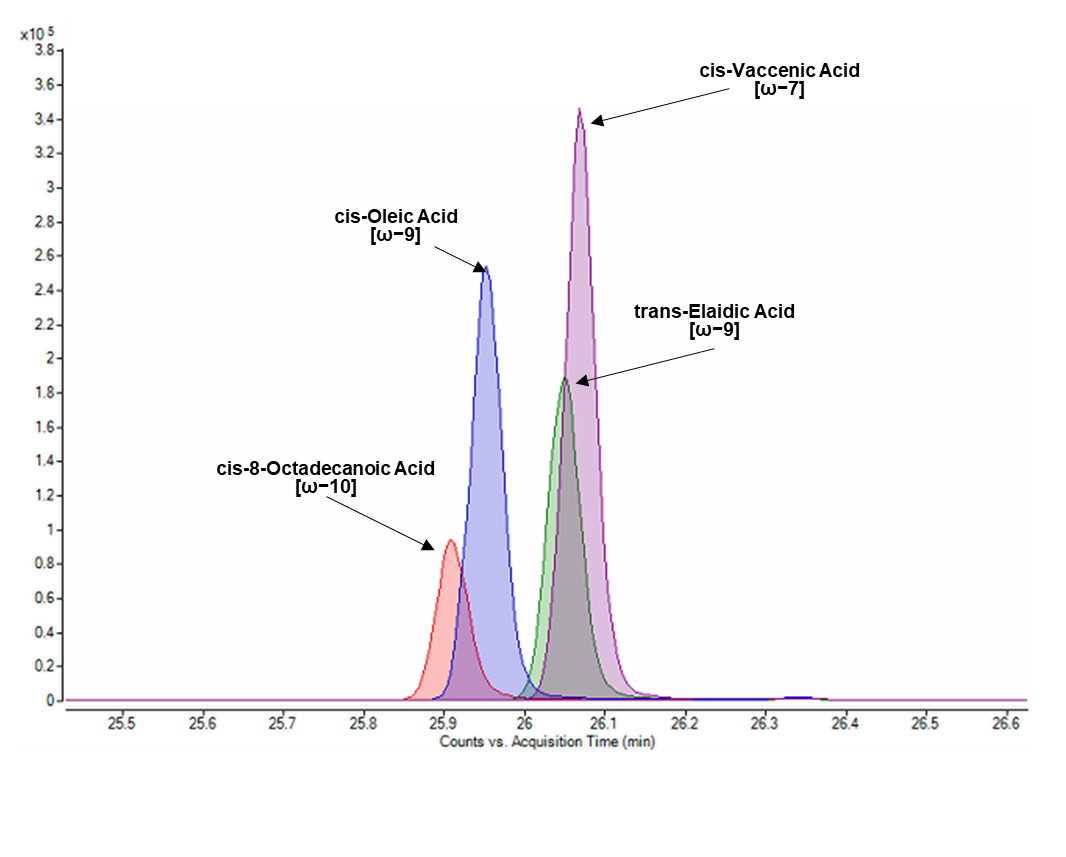


Figure S1: Separation of cis-8-octadecanoic acid, cis-oleic acid, trans-elaidic acid and cis-vaccenic acid derivatized with PFB and subsequently measured by GC-APCI-MS. A separation of the cis/trans isomers as well as a separation of the fatty acids from a difference of the double bond position of at least 2 carbon atoms could be detected.


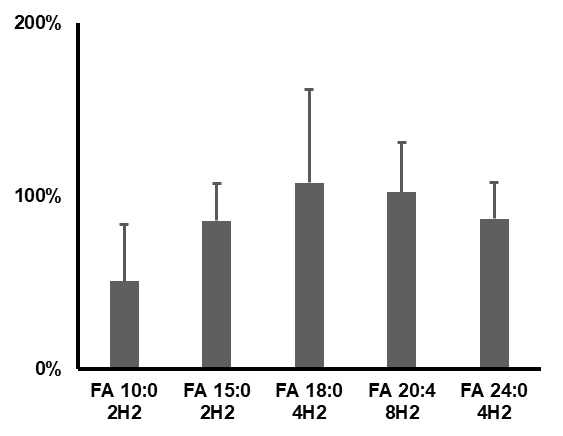


Figure S2: Recovery of the internal standard using the described extraction method (n=5). Recovery was determined by comparing the area of the internal standards spiked to a blank extraction at the beginning and at the end of the extraction.


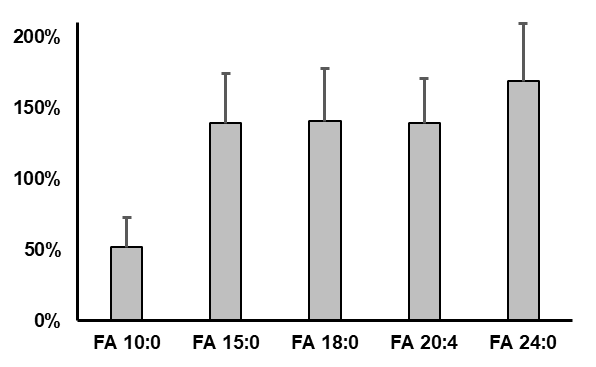


Figure S3: Matrix effect on the internal standards in a series of cell samples (n=40). For the determination of the matrix effect, a series of cell samples were analyzed and the areas of the internal standards were compared to the area of a reference measurement with the same concentration (2,000 nM).
